# Development of *Kaptive* databases for *Vibrio parahaemolyticus* O- and K-antigen genotyping

**DOI:** 10.1099/mgen.0.001007

**Published:** 2023-05-02

**Authors:** Linda van der Graaf-van Bloois, Hongyou Chen, Jaap A. Wagenaar, Aldert L. Zomer

**Affiliations:** ^1^​ Department of Biomolecular Health Sciences, Faculty of Veterinary Medicine, Utrecht University, Utrecht, The Netherlands; ^2^​ WHO Collaborating Centre for Reference and Research on Campylobacter and Antimicrobial Resistance from a One Health Perspective/WOAH Reference Laboratory for Campylobacteriosis, Utrecht, The Netherlands; ^3^​ Shanghai Municipal Center for Disease Control and Prevention, Shanghai, PR China; ^4^​ Wageningen Bioveterinary Research, Lelystad, The Netherlands

**Keywords:** K-locus, O-locus, serotyping, *Vibrio parahaemolyticus*, whole-genome sequencing, genotyping

## Abstract

*
Vibrio parahaemolyticus* is an important food-borne human pathogen and presents immunogenic surface polysaccharides, which can be used to distinguish problematic and disease-causing lineages. *V. parahaemolyticus* is divided in 16 O-serotypes (O-antigen) and 71 K-serotypes (K-antigen). Agglutination tests are still the gold standard for serotyping, but many *V. parahaemolyticus* isolates are not typable by agglutination. An alternative for agglutination tests is genotyping using whole-genome sequencing data, by which K- and O- genotypes have been curated and identified previously for other clinically relevant organisms with the software tool *Kaptive*. In this study, *V. parahaemolyticus* isolates were serotyped and sequenced, and all known and several novel O- and K-loci were identified. We developed *Kaptive* databases for all O- and K-loci after manual curation of the loci. In our study, we could genotype the O- and K-loci of 98 and 93 % of the genomes, respectively, with a *Kaptive* confidence score higher than ‘none’. The newly developed *Kaptive* databases with the identified *V. parahaemolyticus* O- and K-loci can be used to identify the O- and K-genotypes of *V. parahaemolyticus* isolates from genome sequences.

## Data Summary

The sequence data of the *Vibrio parahaemolyticus* isolates have been deposited at the European Nucleotide Archive (ENA) under projects PRJEB39490 and PRJNA483379, and the accession numbers are listed in the (Table S1, available with the online version of this article).

**Impact Statement**

Serotyping is one of the most used methods for identification and epidemiology of pathogens. Agglutination tests are still the gold standard for serotyping *Vibrio parahaemolyticus* isolates; however, these tests are costly and time consuming. An alternative is using genome sequences for molecular epidemiology through sequence typing; however, the sequence type does not always translate directly to serotype. An alternative for agglutination tests is genotyping; predicting the serotype from whole-genome sequencing data. In our study, we manually curated genotyping databases for *V. parahaemolyticus* for all known and several novel O- and K-loci. By using the genotyping tool *Kaptive*, we could genotype the O- and K-loci of 98 and 93 % of the genomes, respectively, with a *Kaptive* confidence score higher than ‘none’. The newly developed *Kaptive* databases with the identified *V. parahaemolyticus* O- and K-loci can be used to identify the O- and K-genotypes of *V. parahaemolyticus* isolates from genome sequences.

## Introduction

*Vibrio parahaemolyticus* is an important food-borne human pathogen that naturally inhabits marine environments worldwide, and can cause acute gastroenteritis and septicaemia in humans [1, 2]. *V. parahaemolyticus* is typically serotyped on the basis of its heat-stable O-antigenic polysaccharide (O antigen) and its capsular polysaccharide (K antigen), which are found on the bacterial surface, and is classified into 16 O-serotypes and 71 K-serotypes.

Serotyping of *V. parahaemolyticus* is important for pathogen detection and epidemiological surveillance. Many *V. parahaemolyticus* serotypes have been identified as pandemic clones, and certain serotypes, for example O3:K6, O1:KUT and O4:K68, are generally considered to be more virulent than others [3, 4]. Agglutination tests are the gold standard for *V. parahaemolyticus* serotyping, but frequently *V. parahaemolyticus* strains are not agglutinated by any diagnostic antisera against known serotypes and are, therefore, not typable [3, 5–7]. For several pathogenic bacterial species, e.g. several pathogenic *E. coli* serogroups [8] and *Vibrio cholerae* serogroup O1 and O139 [9, 10], PCR methods have been developed to detect the specific serogroups, but no PCR methods are available for *V. parahaemolyticus* genotyping.

The software tool *Kaptive* was developed for rapid O- and K-loci typing of *Klebsiella* strains from whole-genome sequences based on blast analysis of assemblies [11]. This tool includes an option to use other, self-created databases. The genomic regions associated with the somatic synthesis locus (O-locus) and capsule synthesis locus (K-locus) for *V. parahaemolyticus* are both generally flanked with known core genes (the O-locus between genes *dgkA* and *gmhD*, and the K-locus between genes *gmhD* and *rjg*) [2, 12, 13], which makes it possible to locate and extract the O- and K-loci nucleotide sequences from whole-genome sequencing (WGS) data of *V. parahaemolyticus* strains. Recently, the algorithm VPsero was developed, which identified serogroup-specific genes as markers *in silico*, but only marker genes for 12 O- and 43 K-serotypes were covered [14].

The aim of this study was to identify all 16 O- and 71 K-loci of *V. parahaemolyticus* by serotyping, sequencing and analysing in-house isolates, and investigating public data of serotyped strains, and manually curate reference databases for O- and K-loci, which can be used to determine the O- and K-genotypes of *V. parahaemolyticus* strains from WGS data.

## Methods

### Culturing and serotyping of *V. parahaemolyticus* isolates

In this study, 86 *V *. *parahaemolyticus* isolates were obtained from the Shanghai Diarrhea Comprehensive Surveillance System (SDCS). Isolates were retrieved from −72 °C and incubated on tryptone soya agar (TSA) with 3 % sodium chloride at 35 °C for 24 h. Serotyping was performed by slide agglutination (Denka Seiken), according to the manufacturer’s instructions. The Denka Seiken kit used has sera for 11 O-serotypes and 65 K-serotypes (https://www.denka.co.jp/eng/pdf/product/medical/detail/00325/bacterial-handbook4th.pdf, page 22). There are 16 O- and 71 K-serotypes known for *V. parahaemolyticus* and sera for multiple O-serotypes (O12–O16) and multiple K-serotypes (K2, K14, K16, K27, K35, K62) are not commercially available and, therefore, were not tested.

### Genomic datasets of *V. parahaemolyticus*

For WGS of the 86 *V *. *parahaemolyticus* isolates, DNA was extracted with the TIANamp Bacteria DNA kit (Tiagen Biotech) according to the manufacturer’s recommendations, and DNA quality control was performed using agarose gel electrophoresis and the Qubit dsDNA HS assay kit. DNA libraries and DNA nanoballs (DNB) were constructed on the BGISP-100 platform (WuHan MGI Tech) with input of 150 ng DNA. Single-end reads of 50 bp were generated with a BGISEQ-50 sequencer (WuHan MGI Tech) and assembled with SPAdes v3.12 [15]. Contigs smaller than 200 bp and with a coverage lower than 10 were removed, and genome quality was assessed using CheckM v1.1.2 [16] for completeness (>95 %) and contamination (<5 %). All sequence reads of strains sequenced in this study are available under projects PRJEB39490 and PRJNA483379 of the European Nucleotide Archive (ENA) short-read archive.

Two datasets of publicly available *V. parahaemolyticus* genomes were included, consisting of 775 genomes from the National Center for Biotechnology Information (NCBI) GenBank (access date: 14 December 2017) and 1498 genomes from the PATRIC database [17] (access date: 23 July 2020). Taxonomic classification of all genomes was performed by *in silico* 16S DNA analysis with spingo v1.3 [18] and genomes with ambiguous 16S results were classified as *V. parahaemolyticus* using KmerFinder v3.2 [19].

### Development of *Kaptive* databases

#### Identification of O-loci

The O-locus of *V. parahaemolyticus* has been defined as being located between *dgkA* and *gmhD* genes, which encode a diacylglycerol kinase and an epimerase, respectively [2]. With blastn (minimum coverage of 80 % and minimum identity of 80 %), the flanking genes of the O-region, *dgkA* (accession no. AOG18133) and *gmhD* (accession no. AOG18159), were searched in all the genome sequences. The O-loci sequences were extracted from these sequences manually.

#### Identification of K-loci

The K-locus of *V. parahaemolyticus* has been defined as being located between gene *gmhD* on one side, which encodes an epimerase, and at the other side gene *rjg*, encoding a metallo-hydrolase [13]. With blastn (minimum coverage of 80 % and minimum identity of 80%), the flanking genes of the K-region, *gmhD* (accession no. AOG18159) and *rjg* (accession no. QEQ70639), were located in all 1622 genome sequences. In some cases where the *rjg* gene was not found, gene *ugd* (accession no. WP_069500066) or gene *gtaB* (accession no. EHK2853238) was selected as the flanking gene. The K-loci sequences were extracted from these sequences manually.

#### Development of the *Kaptive* databases

The extracted O- and K-loci sequences were annotated using Prokka v1.13 [20]. For annotating the loci, a custom database was used that was built with the annotations of the previously described loci of *V. parahaemolyticus* [1, 2]. Genes of the O- and K-loci were clustered using Roary v3.12.0 with a 97 and 90% cut-off, respectively, on amino acid identity [21]. The Roary gene presence–absence table was used to create gene presence–absence clusters of the O- and K-loci, and the unique gene presence–absence loci were linked to the known serotypes of the genomes.

To identify insertion sequence (IS) elements in the sequences, ISEScan v1.7.2.3 [22] was used with default parameters. The annotations of identified IS elements with an *E* value <1×10^−15^ were removed from the GenBank files of the *Kaptive* databases.

For the identified O- and K-loci, the nomenclature of the *Klebsiella* capsule synthesis loci [11] was used; each distinct O- and K-locus was designated as OL (O-locus) and KL (K-locus), followed by an unique number. The O- and K-loci of known serotypes were assigned with the same number as the corresponding O- and K-serotypes, e.g. O-serotype 1 is encoded by the OL1 locus, etc. Variants of loci were assigned if they contained a maximum of two genes different by insertion or deletion, and were given the suffix −1. The O- and K-loci with unknown serotypes and more than two genes different from known loci were assigned as new loci with numbers starting from 101 (e.g. OL101 and KL101). Eight gene patterns of K-untypable (KUT) strains were found and these patterns are assigned in the K-loci database as KLUT (K-locus untypable) followed by subsequent numbers 1–8.

For each locus, one genome was selected as a reference and the nucleotide sequences of the O- and K-loci of the selected genomes were added to the *Kaptive* database file and curated manually. The newly developed *Kaptive* databases contain a total of 18 O-loci and 133 K-loci. The *V. parahaemolyticus* O-locus and K-locus *Kaptive* databases are available on the following GitHub page – https://github.com/aldertzomer/vibrio_parahaemolyticus_genomoserotyping – and have been added to *Kaptive* Web –

https://kaptive-web.erc.monash.edu/

### Genome phylogeny of genotypes

The reference genomes of the identified O- and K-loci were aligned using Parsnp v1.2 [23], recombination regions were filtered using Gubbins v2.3.4 [24] and the tree was built with FastTree v2.1.8 [25]. The tree was visualized with iTOL v6.5.4 [26] and multilocus sequence typing (MLST) was performed *in silico* with the MLST tool mlst v2.10 (https://github.com/tseemann/mlst) using PubMLST typing schemes [27]. Gene cluster comparisons of the O- and K-loci were made with Clinker v0.0.37 [28].

### Performance of *Kaptive* databases compared to VPsero

Of the total set of 852 selected genomes from GenBank (*n*=766) and in-house sequenced genomes (*n*=86), 446 genomes had a known O-serotype and 301 genomes had a known K-serotype, whereas the PATRIC genomes had all unknown O- and K-loci serotypes. The genomes with known serotypes were used as a reference to test the performances of the newly developed *Kaptive* databases and VPsero [14].

Performance of the *Kaptive* databases was tested and compared with VPsero by using the VPsero sequence data collection containing 418 sequences, deposited into CNGB (China National GeneBank) Sequence Archive under project CNP0000343, as described by Bian *et al*. [14]. The performance of the *Kaptive* databases was tested by using the *Kaptive* tool v2.0.4 with the commandline (https://github.com/katholt/Kaptive/), using default parameters. The performance of VPsero was tested by using VPsero (https://github.com/shengzheBian/VPsero) with default parameters.

## Results and discussion

### Development of *Kaptive* databases for *V. parahaemolyticus* O- and K-loci

The study started with a set of 2330 *V *. *parahaemolyticus* genomes, consisting of 86 genomes sequenced in this study, 776 genomes downloaded from NCBI GenBank and 1498 from the PATRIC database. The genomes that were *in silico* identified as *V. parahaemolyticus* with 16S and KmerFinder analysis, and with CheckM results of >95 % completeness and <5 % contamination, were included. For the genomes downloaded from NCBI GenBank and the PATRIC database, only genomes that contained the complete loci of either the O- or K-locus on one contig were included. This selection resulted with a total set of 1622 genomes, consisting of 86 in-house sequenced strains, 766 from GenBank and 770 genomes from PATRIC database. No isolate nor genome was available for K-serotypes 26 and 71 and, therefore, these serotypes were not included in the database.

For each O-serotype, unique loci were identified, except for serotype O3 and O13 genomes, since the loci of these serotypes contained the same genes [1]. Therefore, loci of serotype O3 and O13 could not be distinguished based on gene presence and are assigned in the *Kaptive* O-database as ‘O3_or_O13’. It is possible that a second cluster modifies the O-antigen, similar to what has been described for *Shigella* [29]; however, an O-modifying locus has not been described in the literature for *V. parahaemolyticus* O3 or O13. Eight gene patterns of KUT strains previously described were found and these patterns were assigned in the *Kaptive* database as KLUT, followed by subsequent numbers 1–8 (Table S1).

For both O- and K-loci, not all gene patterns could be assigned to specific serotypes, because these gene patterns were found in genomes with unknown serotypes from NCBI and PATRIC databases. These unknown gene patterns were assigned for O-loci as OL101 and for K-loci as KL101–KL157 (Table S1). For some O- and K-serotypes, variants in the loci gene patterns by insertion or deletion of a maximum of two genes were found and, therefore, these serotypes have two gene patterns included in the database, distinguished with the addition of ‘−1’ to the locus name (OL1, OL4, OL7, KL20, KL30, KL68, KLUT4) (Figs S1 and S2).

**Fig. 1.**
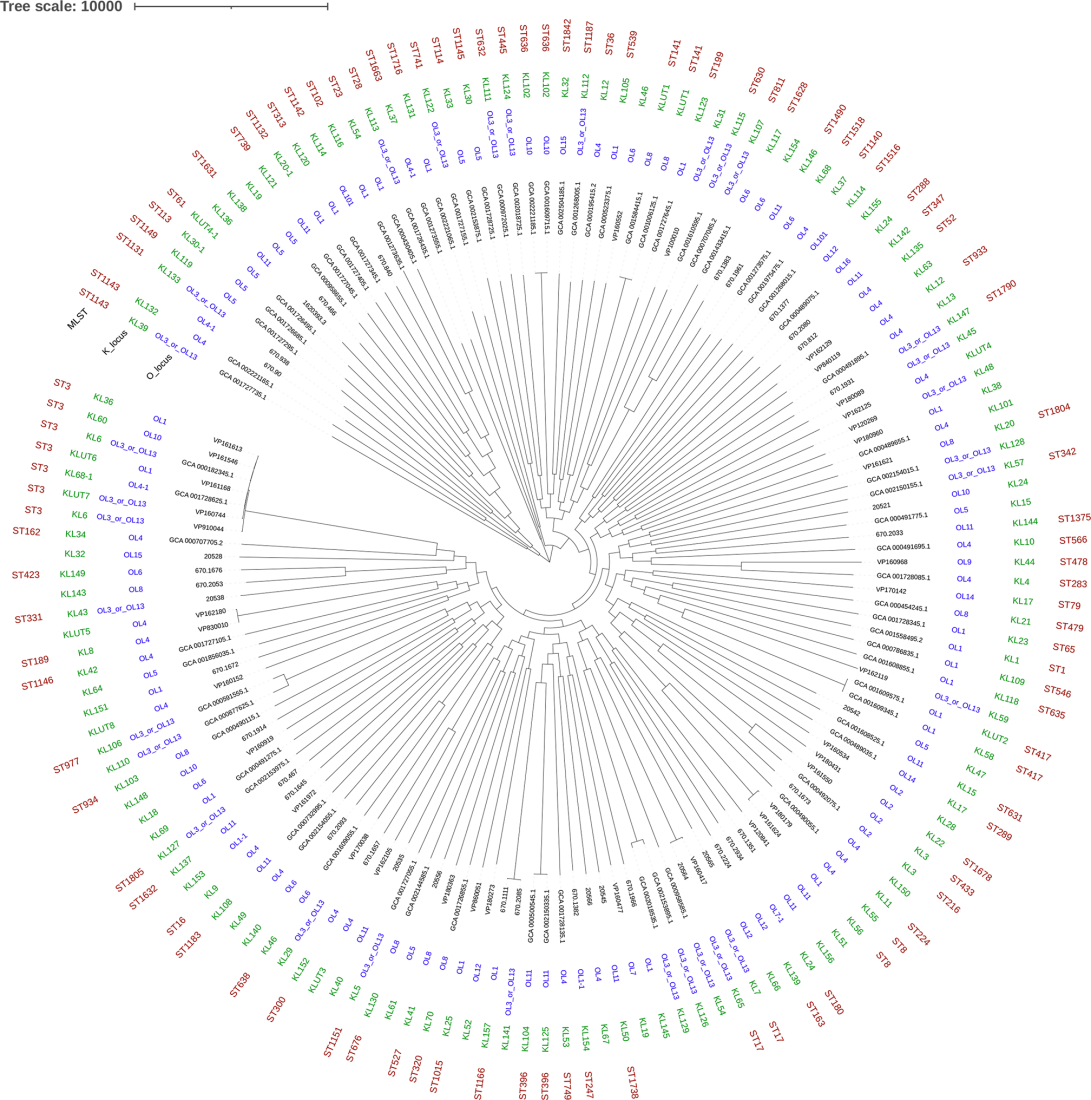
Phylogenetic tree based on core-genome analysis of O- and K-loci reference genomes, including the O- and K-loci, and MLST sequence types. The scale bar represents recombination-filtered point mutations.

The newly developed *Kaptive* databases contain a total of 18 O-loci and 133 K-loci. The selected genomes that were used as references for each O- and K-loci are shown in Table S1. Examination of the phylogenetic tree of the reference isolates showed the O- and K-loci are not associated with MLST sequence types, e.g. six different K-loci and four different O-loci are found in ST3 reference isolates (Fig. 1).

### Performance of *Kaptive* databases

#### Genome collection of this study

Of the total set of 852 selected genomes from GenBank (*n*=766) and in-house sequenced genomes (*n*=86), 446 genomes had a known O-serotype and 301 genomes had a known K-serotype, whereas the PATRIC genomes had all unknown O- and K-loci serotypes. Of the 446 known O-serotypes, 406 (91 %) O-genotypes were correctly identified with the *Kaptive* databases with a confidence score higher than ‘none’ (Table S1) and 272 (90 %) of the 301 known K-serotypes were correctly identified with the *Kaptive* databases with a confidence score higher than none (Table S1).

**Fig. 2.**
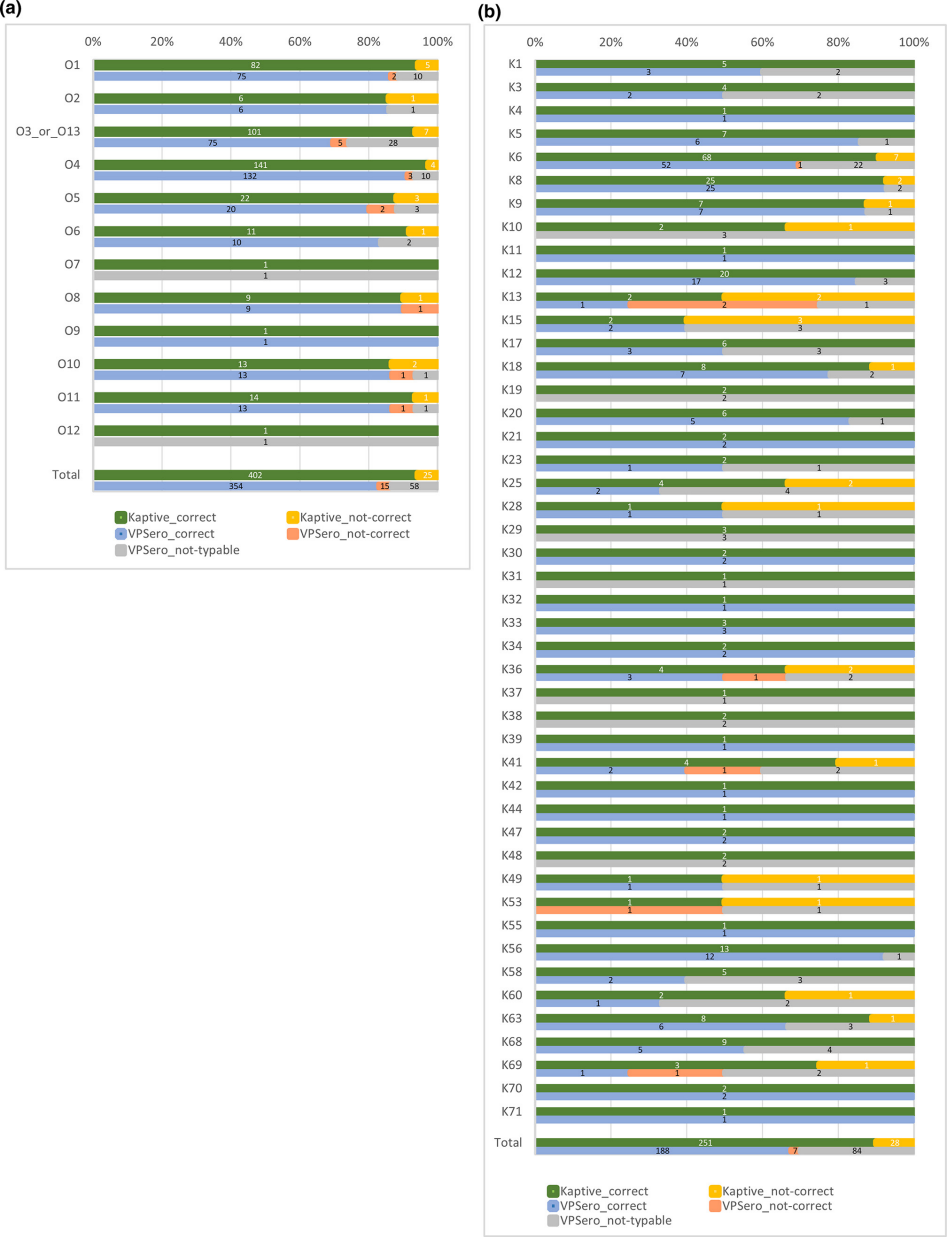
Performance of the *Kaptive* database and VPsero on genomes with known (**a**) O- and (**b**) K-serotypes. In green are the percentages of genomes correctly genotyped with *Kaptive* databases, in orange the percentages of genomes incorrectly genotyped with *Kaptive* databases, in blue the percentages of genomes correctly genotyped with VPsero, in red the percentages of genomes incorrectly genotyped with VPsero, and in grey the percentages of VPsero not-typable genomes. The numbers in the bars represent the numbers of genomes.

The tool VPsero was developed for 46 K- and 12 O-serotypes [14], and covers part of the known 71 K-serotypes and 16 O-serotypes. The performance of VPsero was tested with a set of 427 genomes for O-genotyping and 279 genomes for K-genotyping from our genome collection, consisting of genomes with known serotypes of the 12 and 46 O- and K-serotypes, respectively, which are included in VPsero. Of this set, 354 (83 %) out of 427 genomes with known O-serotypes were correctly identified with VPsero (Fig. 2a, Table S1) and 188 (67 %) out of 279 genomes with known K-serotypes (Fig. 2b, Table S1). The newly developed *Kaptive* databases outcompete VPsero with 402/427 (94 %) correctly identified O-genotype genomes and 251/279 (90 %) correctly identified K-genotype genomes (Fig. 2a, b, Table S1).

#### Sequence collection of VPsero

In VPsero, intact LPS (lipopolysaccharide) and CPS (capsular polysaccharide) gene clusters (LPS/CPSgcs) were identified and extracted, and deposited in the CNGB Sequence Archive under project accession number CNP0000343. These gene clusters did not contain the full O-loci of the newly developed O-genotype *Kaptive* database and, therefore, this sequence data collection could not be used to test the performance of the O-genotype *Kaptive* database; the K-serotype loci were available however. The LPS/CPSgcs were tested with both *Kaptive* K-database and VPsero, and results are listed in Table S1. One K58 gene cluster was identified as K6 by both tested tools, possibly containing a wrong serotype in the CNGB database. Of the remaining 56 gene clusters with known K-serotypes, 7 were not typable by VPsero and 2 were mis-identified by *Kaptive* databases.

The mis-identified O- and K-genotypes from our collection were all genomes downloaded from NCBI. Since we only have the genomes and not the strains, we cannot check whether these strains were serotyped correctly. For the O-genotype, out of 446 known O-serotypes, 25 genomes (5.6 %) were genotyped incorrectly, of which 1 genome (0.2 %) had a confidence score of none. Of all genomes, 15 out of 852 genomes (1.8 %) had a O-locus confidence score of none. For the K-genotype, out of 301 known K-serotypes, 29 genomes (9.6 %) were incorrectly genotyped. Of these, 20 genomes (6.6 %) have a ‘good/very high’ confidence score in *Kaptive*, with near 100 % ID and coverage, and 7 genomes have a score of none (2.3 %). Of all genomes, 60 out of 852 (7.0 %) genomes had a K-locus confidence score of none. The confidence score of none suggests that the locus is not complete, or that the cluster is not present in the *Kaptive* database, and may also be due to false positive agglutination results.

O-serotype O14 has been recently described [2] and only one genome with this O-genotype is available. In our dataset of 852 genomes, 13 NCBI genomes with serotype O5 are identified with the *Kaptive* database as the new genotype O14. It is very likely that these genomes are mis-genotyped, because the serum for serotype O14 was not available when these genomes were serotyped.

For several K-serotypes, only one genome sequence was available and we could not determine whether there was variation in the gene content of these K-loci . Furthermore, several of the genomes sequenced in this study were sequenced with a 50 bp single-end BGI sequencer, resulting in a higher number of short contigs; therefore, for several of these genomes, the K-locus was not assembled on one single contig. For these genomes, the contigs with flanking genes were selected and concatenated manually. The contiguity of the loci of the concatenated contigs was checked with Bandage v0.9.0 [30]. If more *V. parahaemolyticus* genome sequences with closed K-loci become available, the *Kaptive* K-serotype database will be updated with the closed K-locus sequences of these K-serotypes.

### Conclusion

The *Kaptive* databases developed in this study with the identified 16 O- and 71 K-loci can be used to identify the O- and K-genotypes of *V. parahaemolyticus* isolates from WGS data. The variation of K-antigen loci is much higher than expected, as we identified 57 new K-locus variants.

## Supplementary Data

Supplementary material 1

Supplementary material 2
