# Supplementary material for: Development of Kaptive databases for Vibrio parahaemolyticus O- and K-antigen genotyping
Source: Microb Genom. 2023 May 2;9(5):mgen001007. doi: 10.1099/mgen.0.001007 (PMC10272883; doi:10.1099/mgen.0.001007)
Supplement: Supplementary material 2 [file mgen-9-1007-s002.pdf]

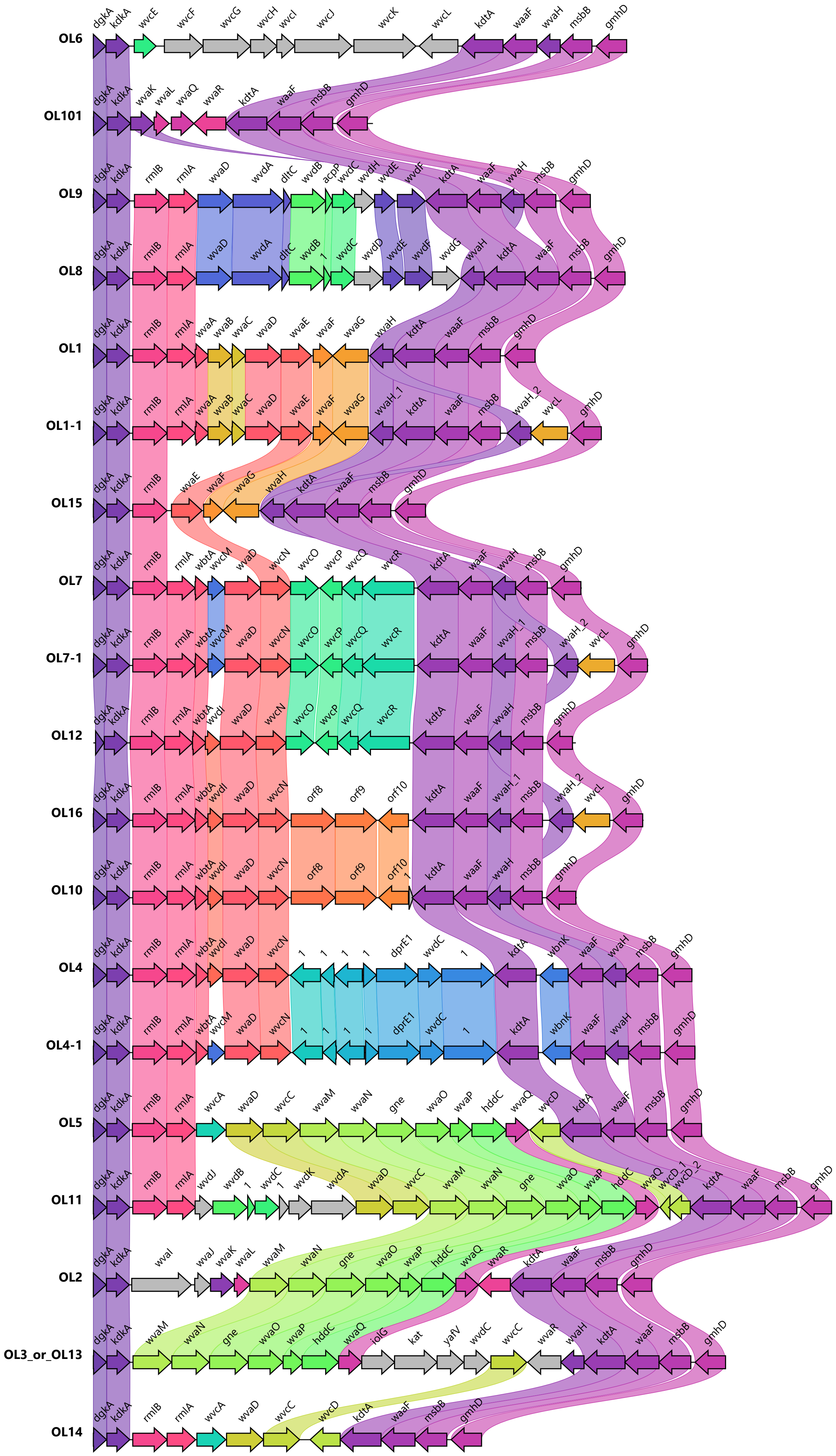

Supplemental Figure 1.
Gene cluster comparisons of the identified O-loci. All similar gene homologs have been assigned an unique color and are linked with similar color boxes. Hypothetical proteins are assigned with an “1”.

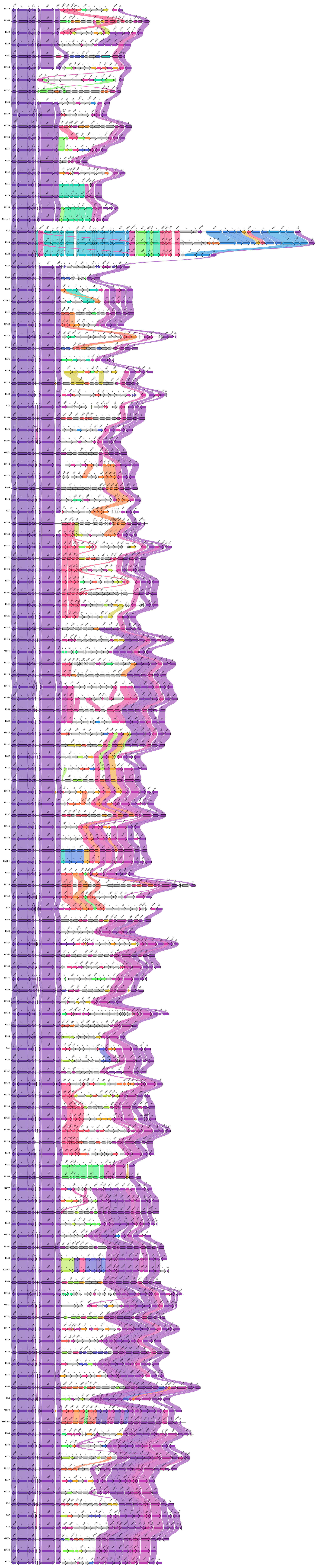

Supplemental Figure 2.
Gene cluster comparisons of the identified K-loci. All similar gene homologs have been assigned an unique color and are linked with similar color boxes. Hypothetical proteins are assigned with an "1".
